# Clinical and demographic characteristics of chronic kidney disease patients in a tertiary facility in Ghana

**DOI:** 10.11604/pamj.2014.18.274.4192

**Published:** 2014-08-04

**Authors:** Yaw Ampem Amoako, Dennis Odai Laryea, George Bedu-Addo, Henry Andoh, Yaw Asante Awuku

**Affiliations:** 1 Department of Medicine, Komfo Anokye Teaching Hospital, Kumasi, Ghana; 2 Public Health Unit, Komfo Anokye Teaching Hospital, Kumasi, Ghana; 3 Central Regional Hospital, Cape Coast, Ghana

**Keywords:** Chronic kidney disease, dialysis, developing world, Ghana

## Abstract

**Introduction:**

Chronic kidney disease (CKD) has emerged as a public health challenge in countries around the world. The cost of management of CKD is enormous and unaffordable to most patients in the developing world. There is a dearth of data on characteristics of Ghanaian CKD patients at presentation.

**Methods:**

This was a prospective cross sectional study of CKD patients during their first visit to the renal clinic of a tertiary hospital adult renal service. Following informed consent, a questionnaire was used to gather demographic, anthropometric and clinical details of patients. Laboratory data of patients were also collected and analysed.

**Results:**

The majority (64.5%) of 203 participants were male. Most were less than 60 years old and about one third were unemployed. Across all age groups stage 5 disease was the commonest presentation; however only 4.3% could afford to initiate haemodialysis. The mean number of dialysis sessions was 12.4 (range 6-18). Chronic glomerulonephritis (33%), hypertension (21.2%) and diabetes mellitus (22.2%) were found to be the leading causes of CKD. Common complications of CKD at presentation included anaemia (86.7%), pulmonary oedema (31%), high blood pressure (55%), and infection.

**Conclusion:**

Early detection of CKD and institution of measures to slow disease progression are to be encouraged. There is the need to make renal replacement therapy increasingly accessible and affordable to patients.

## Introduction

The kidneys play a central role in fluid, electrolyte and acid base homeostasis in humans. In chronic kidney disease (CKD), irreversible damage results in an inability of the kidneys to perform its vital homeostatic, excretory and synthetic functions. CKD is the presence of kidney damage, manifested by abnormal albumin excretion or decreased kidney function that lasts longer than three months as quantified by measured or estimated glomerular filtration rate (eGFR) [1]. Progressive renal disease usually leads to the common end point - end stage kidney disease (ESKD) - of a shrunken, fibrotic kidney. The cost for renal replacement services for ESKD is enormous. In the UK and Italy, the 0.02% - 0.06% ESKD population account for an estimated 0.7%-1.8% of the health service budget [1]. In the United States, the expenditure on ESKD was estimated as US $28 billion in 2010 [2]. CKD affects between 5-15% of the adult population in the developed world [3–5]. In Africa, CKD is estimated to affect about 10.4% of some populations [6, 7] making it a significant public health issue. It has been found to account for 8-10% and 5% of medical admissions in Nigeria [8, 9] and Ghana [10] respectively. The risk factors for CKD abound in the sub - Saharan African population. Osafo [11] and colleagues found a prevalence of 46.9% among hypertensives in a Ghanaian outpatient setting, similar to the findings from an earlier review of autopsy data [12]. In Burkina Faso [13], 44% of hospitalised hypertensives had chronic renal failure. Chronic glomerulonephritis remains an important cause of CKD in tropical Africa [9, 14–16]. Diabetes mellitus and HIV infection are other important contributors to CKD burden [9, 15]. The National Institutes of Health [17] have recommended that patients with chronic progressive renal insufficiency be referred to a multidisciplinary pre-dialysis team in order to minimize patient morbidity and ensure a smooth transition to dialysis therapy. The pre-dialysis clinic is staffed by a multi-disciplinary team, including nephrologists, pre-dialysis nurses, dieticians, and social workers. Components of the pre-dialysis programme include: efforts to delay CKD progression through control of hypertension and hyperglycaemia; patient education regarding CKD, dialysis modalities, and dietary interventions; correction of metabolic abnormalities; insertion of permanent dialysis access; and timely outpatient dialysis initiation [17]. Timely referral to a pre-dialysis programme has been associated with a decreased risk of adverse patient outcomes at the time of initiation of dialysis [18, 19]. Patients referred to a multidisciplinary pre-dialysis teams are better nourished, demonstrate better metabolic profiles, are less likely to require central venous catheter insertion, and require fewer urgent dialysis starts and hospital admission days at the time of dialysis initiation compared to patients who receive standard care [17, 19]. Patients presenting at the later stages of CKD are more likely to have complications requiring emergency interventions and admission. CKD patients in developing countries tend to present with severe disease and with complications. This puts enormous burden on the health system and the few skilled staff working in it. Although CKD remains an important cause of morbidity and mortality in our hospital, there is limited data on patient characteristics and associated factors at initial assessment by the nephrology team. We set out to determine the pattern and clinical presentation of CKD at the Komfo Anokye Teaching Hospital (KATH) over a one-year period.

## Methods

This prospective cross sectional study was carried out over a 1-year period from June 2011 to May 2012 at the Komfo Anokye Teaching Hospital (KATH). KATH, the second largest hospital in Ghana is located in Kumasi and caters for patients in the middle and northern zones of the country. There are clinical services for both adult and paediatric renal patients. Haemodialysis is the main mode of Renal Replacement Therapy (RRT). The study population was all CKD patients accessing services at the KATH Renal Clinic. Consecutive consenting new CKD patients, 18 years or older were recruited into the study. Informed consent was obtained from all study participants or their legal representatives prior to inclusion in the study. A questionnaire was administered to each patient to obtain the demographic data, anthropometric data, and clinical history. Clinical examination and laboratory findings were also recorded. The height was measured with the patient standing barefooted on flat surface. Blood pressure was measured using IntellisenseTM M3 automatic Blood Pressure monitor (Omron Healthcare Europe BV, Netherlands). The pulse pressure was calculated as the difference between the systolic and diastolic blood pressures. A midstream urine sample was analysed for proteinuria using dipstick testing (DIRUI Industrial Co. Ltd, Changuchun, Jilin 130012 P.R. China) and the degree of proteinuria classified as normal, mild or heavy. Abdominopelvic ultrasonography was done to assess the architecture of the kidneys. The haemoglobin (Hb) level and Hb indices were measured at KATH haematology laboratory with an auto analyser (Sysmex KS-21N, Sysmex Corp., Japan). Liver function tests (LFT), blood urea nitrogen (BUN) and serum creatinine levels (based on modified Jaffe method) were measured at the KATH biochemistry laboratory using an auto-analyser (BT 3000 PLUS, Biotecnica Instruments S.p.a, Rome, Italy). Estimated glomerular filtration rate (eGFR) was calculated using the Modification of Diet in Renal Disease (MDRD-4) equation and the stage of CKD noted.

**Ethical statement:** Ethical approval for the study was received from the Committee on Human Research Publications and Ethics (CHRPE) of the School of Medical Sciences, Kwame Nkrumah University of Science and Technology and the Komfo Anokye Teaching Hospital.

**Definition of terms:** CKD was defined as the presence of kidney damage, manifested by abnormal albumin excretion or decreased kidney function, quantified by measured or estimated glomerular filtration rate (GFR) that persists for more than three months. In the absence of previous data on eGFR or markers of kidney damage, chronicity was inferred from clinical presumption of kidney disease for >3 months. Proteinuria was defined as normal (urine dipstick negative), mild (urine dipstick reading trace or 1 + ), or heavy (urine dipstick reading greater than or equal to 2 + ). Hypertension was defined as the presence of a persistently elevated systolic blood pressure ≥ 140mmHg and/or diastolic blood pressure ≥ 90mmHg in patients aged 15 years and above, and/or the use of antihypertensive drugs and/or past medical history of hypertension. Diabetes mellitus (DM) was defined as a random blood glucose level of 11.1mmol/L or greater, and/or fasting blood glucose level of 7.0mmol/L or greater, and/or use of insulin or an oral hypoglycaemic agent. Anaemia was defined as haemoglobin (Hb) level < 11 g/dL. Primary cause of renal disease: The determination of the primary cause of renal disease was based on history, physical examination, and laboratory investigations such as ultrasonography, urinalysis, blood chemistry, and serology. Histological documentation of the primary renal disease was not done since renal biopsies were not a part of this study. The diagnosis of chronic glomerulonephritis (CGN) was largely clinical, based on classical symptoms of loin pain, haematuria, proteinuria, and reduced urine output. Reduced kidney size (<9cm) as well as loss of corticomedullary differentiation were utilised as sonographic evidence of chronic glomerulonephritis. HIV-associated nephropathy (HIVAN) was diagnosed if patient was confirmed HIV positive with low CD4 T cell count and had proteinuria, oedema, and normal-sized or enlarged kidneys on ultrasound. Hypertension was noted as the cause of renal disease in cases with documented medical record if hypertension predated kidney disease and also if there was absence of proteinuria, normal renal function indices, and preserved renal sizes in presence of hypertension early in the illness. Diabetic nephropathy (DN) was diagnosed if patient had a long history of DM, evidence of significant proteinuria with presence of other complications of diabetes mellitus and had normal or increased renal sizes on ultrasound.

**Data analysis:** Data was entered into Microsoft Excel 2007 and analysed using Epi Info version 7.1.2.0. Charts were generated with Microsoft excel.

## Results

**Figure 1:**
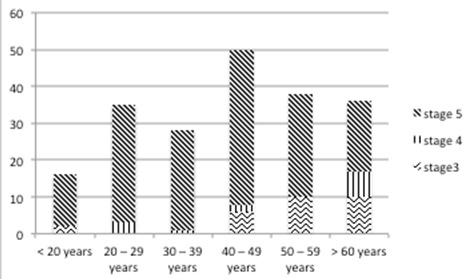
Distribution of chronic kidney disease across age groupings

**Figure 2:**
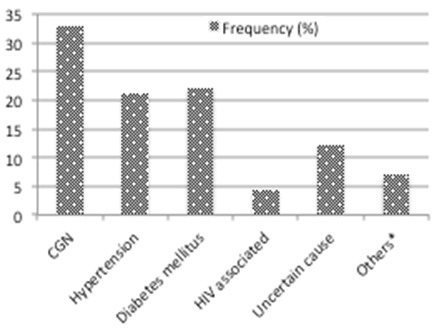
Aetiology of chronic kidney disease in Kumasi, Ghana

A total of 203 participants were recruited for the study. The basic demographic characteristics of the participants are represented in Table 1. Males were in the majority (64.5%) and most patients (87.7%) had received some formal education. The mean age was 43.86 ± 17.84 (range 18 - 85) years. Most participants were less than 60 years. The mean systolic and diastolic blood pressures were 167.9 ± 39.9 (range 100 - 290) and 101.8 ± 24.4 (range 60 - 170) mmHg respectively. The mean arterial pressure (MAP) was 124.6 ± 30.7 (range 70 - 240) mmHg. The median serum creatinine was 1325 (range 136 - 3939) µmol/L (Figure 1). The majority (79.8%) of patients had stage 5 CKD with stage 4 accounting for approximately 6%. Across all age groups, most patients had stage 5 CKD as represented in Figure 1. Only 7 (4.3%) of the 162 stage 5 patients were able to afford haemodialysis. Among this group of patients, the average number of sessions was 12.4 (range 6-18). The commonest clinical features were oedema (88.7%), proteinuria (86.7%), and elevated Blood pressure (85.7%). About 68% had oliguria and pallor was present in 83.2% of respondents (Table 2). Primary aetiology of kidney disease Chronic glomerulonephritis was the cause of CKD in 33.0% of respondents while Hypertension accounted for 21.2% of cases. HIV associated nephropathy and Diabetes mellitus were identified to be the primary cause in 4.4% and 22.2% of cases respectively. The primary aetiology could not be ascertained in about 12.3% of cases. Other identified were Lupus nephritis (1.0%), Autosomal Dominant Polycystic Kidney Disease (1.0%) and Obstructive uropathy (4.4%) (Figure 2, Table 3). The prevalence of anaemia in this cohort was 86.7% with microcytic hypochromic anaemia (58.6%) being the predominant haematologic finding. Approximately fifty five percent (55%) of patients admitted had elevated blood pressure at presentation. Pulmonary oedema and encephalopathy were present in 31% and 8.4% of patients respectively. Pericarditis, gastritis and urinary tract infections were other CKD complications identified.

**Table 1:** Demographic characteristics of respondents

**Parameter**	
**Gender**	**Frequency**
Male	131(64.5)
Female	72(35.5)
Age (mean ± SD) range	
**Religion**	(43.86 ± 17.84) 18 - 85
Christian	162 (79.80)
Muslim	29 (14.29)
None	7 (3.45)
**Educational status**	
Primary	92 (45.3)
Secondary	68 (33.5)
Tertiary	18 (8.9)
None	25(12.3)
**Occupation**	
Farming	35 (17.2)
Teaching	11 (5.4)
Trading	47 (23.2)
Unemployed	77 (37.9)
Others	33 (16.3)
**Marital status**	
Married	113 (55.67)
Single	51 (25.12)
Divorced	18 (8.87)
Widowed	21 (10.34)

**Table 2:** Clinical features of CKD patients at presentation

Characteristics (n = 203)	Percentage (%)
Reduced urine output	67.9
Nocturia	37.9
Haematuria	11.3
Pruritus	9.9
Pallor	83.2
Raised BP	85.7
Pedal oedema	88.7
Proteinuria	86.7
Dipstick Haematuria	25.1
Pericardial rub	4.4

**Table 3:** Complications of CKD at presentation

Diagnosis	Frequency, n (%)
Hypertension	112 (55.2)
Pulmonary oedema	63 (31.0)
Anaemia	176 (86.7%)
Encephalopathy	17 (8.4)
Pericarditis	9 (4.4)
Urinary tract infection	16 (7.9)
Gastritis	8 (3.9)

## Discussion

There was a preponderance of males in this cohort (64.5% vs 35.5%) and this is comparable to similar studies done in Spain [20] and United States of America [21], which also reported a male predominance (60.9% vs. 39.1%) and (61.2% vs. 38.8%) respectively in patients with Chronic Kidney Disease (CKD). Studies in Ghana [15] and Nigeria [22] also report male preponderance (55% vs 45%) and (65.3% vs 34.7%) respectively. The male predominance might be a reflection of the fact that CKD and its risk factors such as hypertension and smoking are commoner in males than females. Differences in the health seeking behaviours of males and females might also play a role in the observed differences in CKD prevalence in the two sexes. The mean age of patients was 43.9 ± 17.8 years, with a peak age between 40 - 49 years. 82.3% were age less than 60 years, the economically active age group. This is similar to the findings from Nigeria [9, 22] and other developing countries [15, 23] but contrasts with that seen in developed countries [24, 25] as depicted in Table 4.

**Table 4:** Comparison of present study with data from developing and developed countries

	Present study	Developing country data (22)	Developed country ANZData registry (25)
Mean age	43.7 ± 17.8	42.6 ± 15.4	65
Peak age group	40– 49	36 – 60	65 - 74
Age prevalence	82.3% < 60 years	86.5% ≤ 60 years	45% > 65 years
Time of presentation	85.8% are CKD stage 4 and 5	CKD stage 4 and 5	25% < 3 months prior to first dialysis

ANZData registry: Australia, New Zealand Data registry; CKD: Chronic Kidney Disease

Several factors may account for the younger age of patients with CKD in the developing world. There is a high prevalence of infections /infestations and these contribute to the development of chronic glomerulonephritis, which is the leading cause of CKD in developing countries [26]. Additionally, inadequate treatment or control of such causes of CKD as hypertension and diabetes mellitus may also be contributory. The second commonest cause of CKD in the tropics is hypertension [26] and hypertension tends to run a more aggressive course in blacks [27]. Diabetic nephropathy occurs at a younger age and is more aggressive in blacks than Caucasian populations [28]. The prevalence of HIV associated nephropathy is also high in the developing world [29]. The primary renal diagnosis could not be ascertained in 12.3% of cases. For those whom the primary kidney disease was known, chronic glomerulonephritis was the most probable cause (33.0%) followed by diabetes mellitus (22.2%) and hypertension (21.2%). Several studies in Ghana [11, 12, 15] and Nigeria [9] have identified chronic glomerulonephritis and hypertension as the commonest causes of CKD. The same is true in other developing countries [23]. In the developed world, diabetes mellitus is the most common cause of CKD [25].

As pertains in other developing countries [22], the majority (85.8%) of the patients in this study presented with advanced CKD (stages 4 and 5) unlike the situation in the developed countries. This late presentation might be partly due to the low detection and treatment/control rates of CKD risk factors like hypertension and diabetes mellitus [12, 30]. The high unemployment rate (37.9%) in this cohort of patients might be a significant contributor to the poor control of blood pressure and late presentation observed in the present study. Other reasons for the late presentation might include the high cost of health care services as well as the use of alternative treatments like spiritualists and traditional healers. In certain communities, patients with generalised body swelling as is the case in CKD are seen as people under a curse and are thus sent to spiritual healers for the reversal of the spell. Such cultural norms may also contribute to the late presentation of patients. The lack of regular CKD screening programmes, inadequate education on CKD and inadequate nephrology services may also be contributing to the late presentation of such patients. The late presentation coupled with the high prevalence among the economically active age group has worrying implications for the socioeconomic wellbeing of individual families and the country as a whole. Renal replacement therapy (RRT) though available at the study site is limited to haemodialysis. There is no capacity for peritoneal dialysis or renal transplantation and the number of skilled personnel is small. RRT is unaffordable to most patients with advanced CKD. The National Health Insurance scheme does limit coverage to only patients with acute kidney injury. In the present study, only 4.3% of patients with stage 5 CKD were able to initiate haemodialysis (HD). The average number of sessions before stoppage (on account of cost) was 12.4 (range 6-18). In a previous report from our centre [15], 50% of 40 patients initiated on HD were able to afford 20 sessions before stopping. In Ibadan, Nigeria, 70% of patients were not able to afford more than 3 sessions of maintenance haemodialysis [31]. The fact that renal replacement therapy is not affordable to most patients requiring such service makes it expedient to institute interventions to prevent the development of ESKD in at risk populations. There is an urgent need to make renal replacement therapy increasing available and affordable to CKD patients to reduce the impact of the disease on society. Public and private sector partnerships may be needed to address the challenge, as the cost involved is enormous for individual patients and their families.

Complications of chronic kidney disease were common in this study. Anaemia was present in 86.7% of patients. The majority (58.6%) had microcytic hypochromic anaemia. The high prevalence of anaemia is consistent with findings from other African studies [32, 33] however; the predominant haematologic finding was normocytic normochromic anaemia in those studies. No iron studies were performed as part of this current study. It is however known that intestinal infestations are common in the study area and this may partly explain the observed blood picture. Also gastrointestinal blood loss, history of haemolysis and hyperparathyroidism may be contributory. Anaemia is a significant contributor to cardiovascular morbidity and mortality in CKD as reduced haemoglobin levels are associated with Left Ventricular Hypertrophy, increased frequency and duration of hospitalization, and reduction in quality of life [34]. The high prevalence of anaemia further increases the cost of treatment. Erythropoietin required for the management of CKD anaemia is available but expensive so most patients cannot afford to use it appropriately.

High mean systolic and diastolic blood pressures were recorded among study participants. This has implications for reducing the rate of cardiovascular disease as there is evidence that blood pressure control reduces the rate of cardiovascular disease in CKD patients [35]. Blood pressure control has also been associated with an attenuation of the rate of GFR decline in those with proteinuria [35]. Guidelines for hypertension treatment in CKD patients recommend pharmacological therapy and lifestyle modification that will achieve a blood pressure goal of less than 130/80 mmHg [36]. This blood pressure target is often difficult to achieve [37]. In the present study, 55% of patients had elevated blood pressure at assessment. Adequate blood pressure control in Blacks is crucial as they have a 5-fold risk of progression from CKD to ESRD when compared with Whites [20, 27].

## Conclusion

Advanced stages of chronic kidney disease are common in patients seeking care at the Komfo Anokye Teaching Hospital. Although majority of affected persons are in the economically active age groups, more than a third of patients are unemployed. Chronic glomerulonephritis (33.0%), hypertension (21.2%) and diabetes mellitus (22.2%) are common causes of chronic kidney disease. Less common aetiologies include Autosomal Dominant Polycystic Kidney Disease, sickle cell nephropathy and obstructive uropathy. The majority of patients have an associated anaemia. Uncontrolled hypertension, pulmonary oedema, and anaemia were common complications during initial assessment of CKD patients. Only a limited proportion of patients are able to afford haemodialysis. It is important to implement appropriate screening programmes to aid early detection of CKD in at risk populations. Early detection and aggressive control of the risk factors for development of CKD are necessary to prevent and reduce the scourge of CKD in resource poor settings where services for renal replacement therapy are not widely available or are unaffordable for most patients requiring such services.
